# Nanoarchitecture‐Integrated Hydrogel Boosts Angiogenesis–Osteogenesis–Neurogenesis Tripling for Infected Bone Fracture Healing

**DOI:** 10.1002/advs.202406439

**Published:** 2024-09-05

**Authors:** Kangkang Zha, Weixian Hu, Yuan Xiong, Shengming Zhang, Meijun Tan, Pengzhen Bu, Yanzhi Zhao, Wenqian Zhang, Ze Lin, Yiqiang Hu, Mohammad‐Ali Shahbazi, Qian Feng, Guohui Liu, Bobin Mi

**Affiliations:** ^1^ Department of Orthopedics Union Hospital Tongji Medical College Huazhong University of Science and Technology Wuhan 430022 China; ^2^ Department of Orthopedics Tongji Hospital Tongji Medical College Huazhong University of Science and Technology Wuhan 430030 China; ^3^ Key Laboratory of Biorheological Science and Technology Ministry of Education College of Bioengineering Chongqing University Chongqing 400044 China; ^4^ Department of Biomaterials and Biomedical Technology University Medical Center Groningen University of Groningen Antonius Deusinglaan 1 Groningen 9713 AV The Netherlands

**Keywords:** angiogenesis, hydrogel, infected fracture, neurogenesis, osteogenesis

## Abstract

Infected fracture healing is a complicated process that includes intricate interactions at the cellular and molecular levels. In addition to angiogenesis and osteogenesis, the significance of neurogenesis in fracture healing has also been recognized in recent years. Here, a nanocomposite hydrogel containing pH‐responsive zinc‐gallium‐humic acids (HAs) nanoparticles is developed. Through the timed release of Zn^2+^, Ga^3+^, and HAs, the hydrogel exhibits potent antibacterial effects and promotes angiogenesis, osteogenesis, and neurogenesis. The enhanced neurogenesis further promotes angiogenesis and osteogenesis, forming a mutually supportive angiogenesis–osteogenesis–neurogenesis cycle at the fracture site. The hydrogel achieves rapid infected fracture healing and improves tissue regeneration in mice. This study proposes a comprehensive treatment approach that combines antibacterial effects with the regulation of tissue regeneration to improve infected fracture healing.

## Graphical abstract

A pH‐responsive nanoarchitecture‐integrated hydrogel is developed to treat drug‐resistant bacteria‐infected fracture healing. Through the controlled release of humic acids, Zn2+, and Ga3+, the hydrogel eradicates bacterial infection, alleviates oxidative stress, and promotes angiogenesis, osteogenesis, and neurogenesis, offering a whole‐course‐repair strategy to accelerate fracture healing in a MRSA‐infected fracture model.

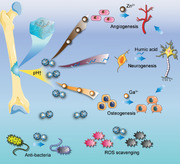

## 1 Introduction

In recent years, the incidence of fracture has gradually increased, among which delayed or nonunion fracture accounts for ≈5–10%.^[^ ^1^ ^]^ Fracture‐related infection is a common complication in open musculoskeletal trauma that can lead to persistent oxidative damage and a prolonged inflammatory course, which greatly impact the process of fracture healing.^[^ ^2^, ^3^ ^]^ Currently, antibiotics are widely used for the treatment of fracture‐related infections. However, it has been demonstrated that overuse of antibiotics exerts negative effects on bone and can lead to drug‐resistant bacteria.^[^ ^4^, ^5^ ^]^ Thus, it is essential to develop novel strategies to eradicate infection, reduce oxidative stress, and promote bone regeneration to facilitate bone fracture healing.

Bone fracture healing is a complex process involving intricate cellular and molecular interactions.^[^ ^6^ ^]^ Currently, bone regeneration strategies mainly focus on enhancing the recruitment and osteogenic differentiation of stem cells and the angiogenic activity of endothelial cells (ECs).^[^ ^7^, ^8^, ^9^ ^]^ Beyond that, bone is a highly innervated tissue, and the role of bone‐nerve crosstalk in fracture repair has also been demonstrated.^[^ ^10^, ^11^ ^]^ For example, Li et al. reported that inhibition of the activity of tropomyosin receptor kinase A (TrkA) resulted in reduced sensory fiber numbers, impaired revascularization, and delayed ossification in the fracture callus, indicating the important role of TrkA‐expressing sensory nerve fibers in fracture healing.^[^ ^12^ ^]^ Thus, the development of a comprehensive strategy to promote bone formation, revascularization, and nerve regeneration will beneficially accelerate fracture healing and improve function.

Recently, several ions have been proposed to participate in the regulation of bone metabolism and show great potential for fracture healing.^[^ ^13^ ^]^ Increasing evidence shows that zinc ion (Zn^2+^) is able to stimulate angiogenesis.^[^ ^14^, ^15^ ^]^ Delivery of Zn^2+^ into the bone defect has been proven to promote angiogenesis and bone regeneration.^[^ ^16^ ^]^ In addition, gallium ion (Ga^3+^) has been proposed to promote osteogenesis and reduce bone resorption.^[^ ^17^ ^]^ Ga‐doped biomaterials represent an efficient approach to enhance bone fracture repair. Moreover, both Zn^2+^ and Ga^3+^ are thought to have excellent antibacterial ability.^[^ ^18^, ^19^ ^]^ These findings promise the great potential of Zn^2+^ and Ga^3+^ as therapeutic agents for infected fracture healing. However, the application of Zn^2+^ and Ga^3+^ is limited by toxicity concerns. To maximize the therapeutic effects and reduce the toxicity of Zn^2+^ and Ga^3+^, it is important to achieve controllable and sustained ion release in vivo according to the different courses of fracture healing.

Humic acids (HAs), natural organic matter that comprises humic substances, can bind various cationic metals and form chelates.^[^ ^20^ ^]^ HAs have pH‐responsive properties, and their solubility is dependent on pH; they are insoluble under acidic conditions and soluble under neutral to alkaline conditions.^[^ ^21^ ^]^ Moreover, HAs exhibit multiple biological activities, including antibacterial, anti‐inflammatory, and antioxidant abilities.^[^ ^22^, ^23^ ^]^ However, the effects of HAs on neurocytes and neurogenesis have not been studied and deserve further investigation. The ability of HAs to bind cationic metals enables their use as Zn^2+^ and Ga^3+^ nanocarriers for bone fracture healing. We suggest that pH sensitivity may enable HAs to release Zn^2+^ and Ga^3+^ according to the pH value in the fracture microenvironment. It is suggested that the physiological microenvironment of the fracture site would turn mildly acidic under conditions of inflammation, blood vessel damage, and bacterial infection at the early stage. With the inflammation resolution and new vessel formation, the pH value gradually increases at the late stage.^[^ ^24^, ^25^ ^]^ When applied in the treatment of infected fractures, Zn‐Ga@HAs NPs can be used to directly kill bacteria at the early stage and promote tissue regeneration in a soluble form at the late stage during fracture healing.

**Scheme 1:**
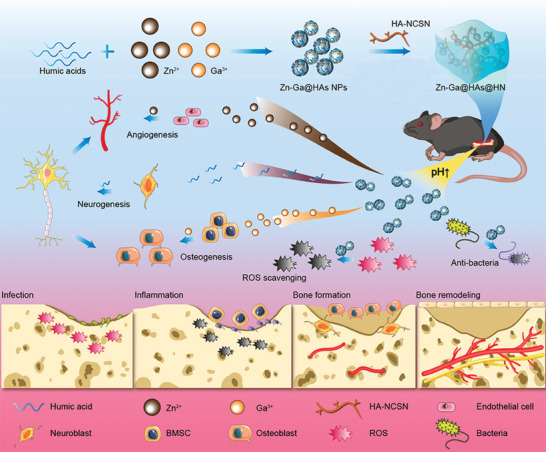
Schematic illustration of the application of the Zn‐Ga@HAs@HN hydrogel in promoting bacteria‐infected bone fracture healing. The Zn‐Ga@HAs NPs were fabricated by chelation of HAs with Zn^2+^ and Ga^3+^. The preparation of the nanoarchitecture‐integrated Zn‐Ga@HAs@HN hydrogel required only one step by mixing the Zn‐Ga@HAs NPs and the polymer HN. The pH sensitivity of Zn‐Ga@HAs NPs enabled the controlled release of Zn^2+^, Ga^3+^, and HAs according to the pH value in the fracture microenvironment. During the early stage, the Zn‐Ga@HAs NPs could eradicate bacterial infection and alleviate oxidative stress. During the later stage, the released Zn^2+^, Ga^3+^, and HAs could boost angiogenesis–osteogenesis–neurogenesis tripling. The angiogenesis and osteogenesis were further enhanced by the newly differentiated neural cells. Concurrently, the Zn‐Ga@HAs@HN hydrogel effectively promoted tissue regeneration and accelerated infected fracture healing.

In the present study, we designed pH‐responsive Zn‐Ga@HAs nanoparticles (NPs) and fabricated a nanoarchitecture‐integrated hydrogel by mixing the NPs with hyaluronic acid‐NCSN (the four major atoms “nitrogen‐carbon‐sulfur‐nitrogen” in the thiourea group) (HA‐NCSN) for infected fracture healing. The nanocomposite hydrogel exhibited great antibacterial activity due to the incorporation of HAs, Zn^2+^, and Ga^3+^. Furthermore, the hydrogel achieved controlled and sustained release of Zn^2+^, Ga^3+^, and HAs, resulting in reduced toxicity and improved angiogenesis, osteogenesis, and neurogenesis, which can meet the requirements for bone regeneration. In this way, the developed hydrogel was able to eradicate bacterial infection and scavenge reactive oxygen species (ROS) in the early stage of fracture healing; during the later stage, the released Zn^2+^, Ga^3+^, and HAs participated in multiple physiological processes and significantly promoted tissue regeneration (**Scheme** **1**). This study proposes a whole‐course repair strategy for infected fracture healing with synergistic regulation of angiogenesis, osteogenesis, and neurogenesis.

## 2 Results

### 2.1 Effects of HAs on Neurogenesis

**Figure 1:**
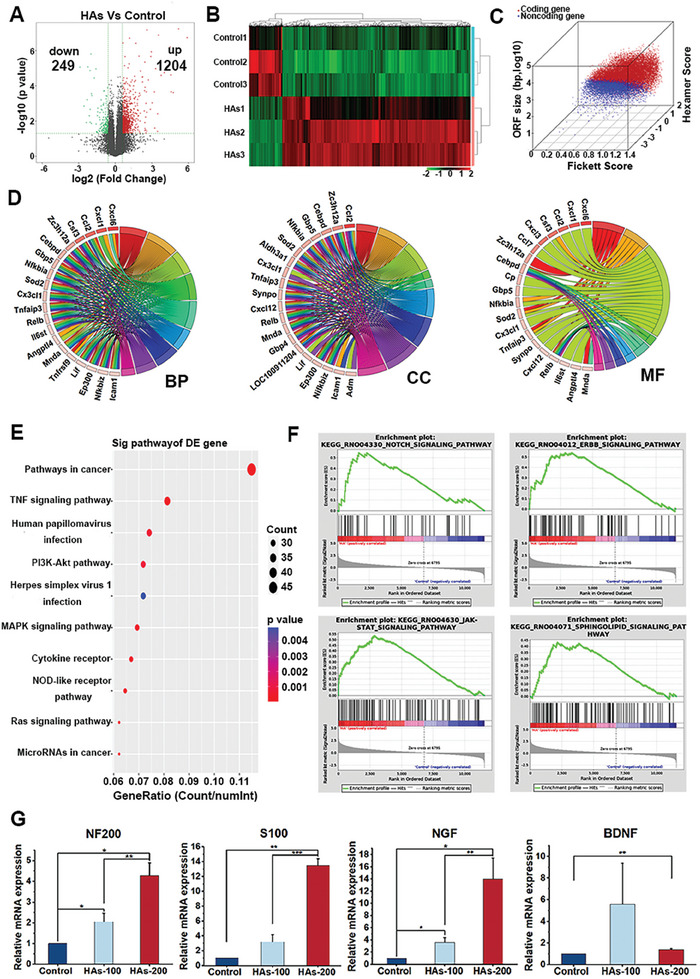
Effects of HAs on neurogenesis. A) Volcano plot showing differentially expressed genes in RSC96 cells treated with HAs. B) Heat map showing the transcription expression profiles of RSC96 cells treated with HAs compared to control cells. C) 3D plot showing the change in coding and noncoding genes after HAs treatment. D) GO classification of genes. E) KEGG enrichment analysis. F) GSEA enrichment analysis. G) qRT‒PCR analysis of the gene expression levels of NF200, S100, NGF, and BDNF in RSC96 cells treated with HAs (100 and 200 µg mL^−1^). (^*^ *p* < 0.05, ^**^ *p *< 0.01, ^***^ *p* < 0.001).

To investigate the impact of HAs on neurogenesis, we performed RNA sequencing (RNA‐Seq) analysis on RSC96 Schwann cells before and after treatment with 200 µg mL^−1^ HAs to identify the changes in gene expression profiles. The samples were quality controlled before analysis and the RNA integrity was satisfactory (Figure S1, Supporting Information). The sequencing results showed that 1453 genes were differentially expressed after HAs treatment (1204 up‐regulated genes and 249 down‐regulated genes) (**Figure** **1**A,B). Figure 1C shows the changes in coding and noncoding transcripts in RSC96 cells after HAs treatment. GO classification of genes revealed that HAs treatment induced the expression of different genes involved in translation, peptide biosynthetic process, cytosolic ribosome, and structural constituent of ribosome (Figure 1D; Figure S2, Supporting Information). KEGG pathway analysis revealed significant changes in genes involved in the NOD‐like receptor signaling pathway, cytokine receptor, MAPK signaling pathway, and PI3K‐Akt signaling pathway (Figure 1E). The results of gene set enrichment analysis (GSEA) further revealed that HAs treatment induced the expression of genes involved in the Notch, ERBB, JAK‐STAT, and sphingolipid signaling pathways (Figure 1F). Then, we detected the effect of HAs on neurogenic gene expression, and the results showed that HAs treatment significantly increased the expression of NF200, S100, NGF, and BDNF in RSC96 cells (Figure 1G).

### 2.2 Synthesis and Characterization of Zn‐Ga@HAs@HN Hydrogel

**Figure 2:**
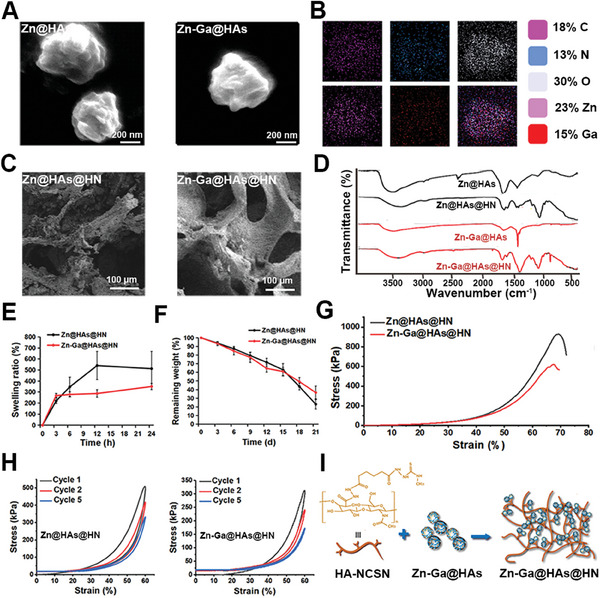
Characterization of the Zn‐Ga@HAs NPs and Zn‐Ga@HA@HN hydrogel. A) SEM images of the Zn@HAs and Zn‐Ga@HAs NPs. B) EDX mapping of Zn‐Ga@HAs NPs. C) SEM images of the Zn@HAs@HN and Zn‐Ga@HAs@HN hydrogels. D) FT‐IR spectra of the hydrogels. E) Swelling ratio of the hydrogels. F) Degradation behavior of the hydrogels in PBS at 37 °C. G) Compression test of the hydrogels. H) Cyclic compression test of the hydrogels. I) Schematic illustration of interactions between Zn‐Ga@HAs NPs and HA‐NCSN in the Zn‐Ga@HAs@HN hydrogel network.

Scanning electron microscopy (SEM) was used to observe the morphology of Zn@HAs and Zn‐Ga@HAs NPs. As shown in **Figure** **2**A, the Zn@HAs and Zn‐Ga@HAs NPs had a diameter of ≈500 nm. Energy dispersive X‐ray (EDX) elemental mapping indicated a homogeneous distribution of Zn and Ga atoms in Zn‐Ga@HAs NPs (Figure 2B). The Zn@HAs and Zn‐Ga@HAs NPs had a similar zeta potential (Figure S3, Supporting Information). The Zn@HAs@HN and Zn‐Ga@HAs@HN hydrogels contained interconnected porous structures, and the pore size was ≈50–200 µm, which facilitated cell perfusion, adhesion, and growth (Figure 2C). Fourier transform infrared spectroscopy (FT‐IR) was performed to characterize the functional groups responsible for gelation (Figure 2D). The observed bonds in the range of 1375 and 1148–947 cm^−1^ in Zn@HAs@HN and Zn‐Ga@HAs@HN hydrogels indicated the formation of coordination bonds between the NPs and HA‐NCSN. Figure 2E shows the swelling behavior of the Zn@HAs@HN and Zn‐Ga@HAs@HN hydrogels. The results indicated that the swelling ratio of the Zn‐Ga@HAs@HN hydrogel (350%) was lower than that of the Zn@HAs@HN hydrogel (512%). As shown in Figure 2F, the degradation rates of the Zn@HAs@HN hydrogel and Zn‐Ga@HAs@HN hydrogel were similar, degrading to 30–40% on day 21. The release of Zn^2+^ from the Zn@HAs@HN hydrogel was faster than that of Ga^3+^ (Figure S4, Supporting Information). The mechanical properties of the hydrogels were also investigated (Figure 2G). The Zn@HAs@HN hydrogel and Zn‐Ga@HAs@HN hydrogel exhibited compression strengths of 927.1 and 620.0 KPa, respectively. After five repeated loading−unloading cycles, the compression strengths of the Zn@HAs@HN hydrogel and Zn‐Ga@HAs@HN hydrogel recovered to ≈50% without rupture (Figure 2H). Figure 2I shows the interactions between Zn‐Ga@HAs NPs and HA‐NCSN in the Zn‐Ga@HAs@HN hydrogel network.

### 2.3 Antibacterial and Antioxidative Abilities of the Zn‐Ga@HAs@HN Hydrogel

**Figure 3:**
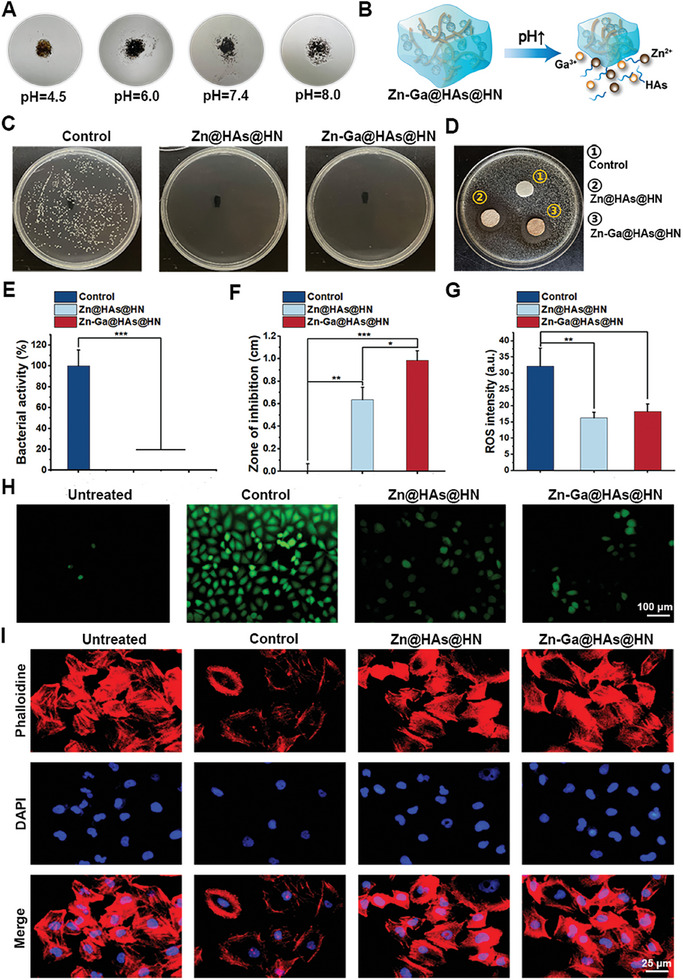
Antibacterial and antioxidative abilities of the Zn‐Ga@HAs@HN hydrogel. A) Degradation of Zn‐Ga@HAs@HN hydrogels immersed in PBS at different pH values for 3 days. B) Schematic illustration of the pH sensitivity of the Zn‐Ga@HAs@HN hydrogel. C,E) MRSA colonies treated with PBS and different hydrogels. D,F) Inhibition zone of the hydrogels against MRSA. G,H) ROS staining by the fluorescent probe DCFH‐DA in HUEVCs after treatment with 500 µm H_2_O_2_ and different hydrogels. I) Cytoskeleton staining images of HUEVCs after treatment with 500 µm H_2_O_2_ and different hydrogels. (^*^ *p* < 0.05, ^**^ *p *< 0.01, ^***^ *p *< 0.001).

As shown in **Figures** **3**A,B, the degradation of the Zn‐Ga@HAs@HN hydrogel accelerated with the increasing of pH value. The antibacterial property of the Zn‐Ga@HAs@HN hydrogel is based on the incorporation of Zn^2+^ and Ga^3+^. Both Zn@HAs@HN and Zn‐Ga@HAs@HN hydrogels exhibited excellent antibacterial capacity, and the inhibition ratios reached ≈99% against MRSA (Figure 3C,E). As expected, evident inhibition zones were observed in the Zn@HAs@HN and Zn‐Ga@HAs@HN groups but not the control groups (Figure 3D). The diameter of the inhibition zone in the Zn‐Ga@HAs@HN group was greater than that in the Zn@HAs@HN groups, indicating the excellent antibacterial activity of Zn‐Ga@HAs@HN hydrogel (Figure 3F). To study the antioxidative ability of the Zn‐Ga@HAs@HN hydrogel, HUEVCs were treated with 500 µm H_2_O_2,_ and Zn@HAs@HN hydrogel or Zn‐Ga@HAs@HN hydrogel or PBS. The results showed that the ROS level significantly increased after treatment with H_2_O_2_, while treatments with Zn@HAs@HN and Zn‐Ga@HAs@HN hydrogels were able to reduce ROS levels in HUEVCs (Figure 3G,H). Cytoskeleton staining of HUEVCs further confirmed that the oxidative stress induced by H_2_O_2_ resulted in cytoskeletal damage in HUEVCs, which was improved after treatment with Zn@HAs@HN and Zn‐Ga@HAs@HN hydrogels (Figure 3I), indicating that the developed hydrogels were able to protect cells from oxidative stress damage.

### 2.4 Angiogenic Properties of the Zn‐Ga@HAs@HN Hydrogel

**Figure 4:**
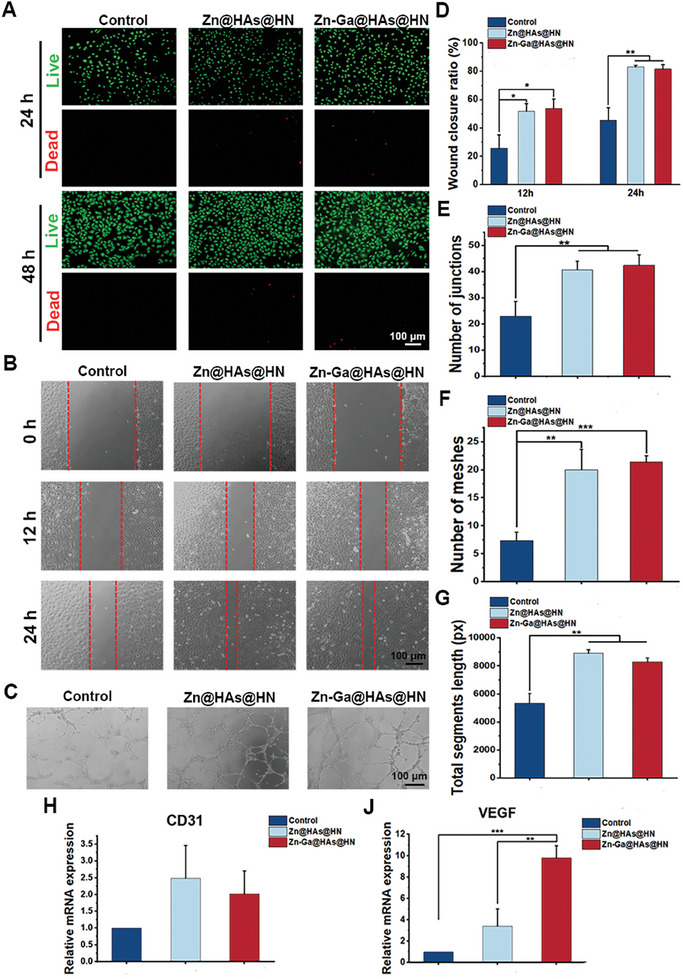
The angiogenic ability of Zn‐Ga@HAs@HN hydrogel. A) Live/dead staining for HUEVCs after treatment with different hydrogels. B,D) Representative images of wound healing and wound closure ratio in HUEVCs treated with different hydrogels at 12 and 24 h. C) Tube formation assay of HUVECs treated with different hydrogels. E–G) Quantitative analysis of the number of junctions, number of meshes, and total segments length. H,J) qRT‒PCR analysis of CD31 and VEGF expression in HUVECs treated with different hydrogels. (^*^ *p* < 0.05, ^**^ *p *< 0.01, ^***^ *p *< 0.001).

Live/dead staining was used to evaluate the cytocompatibility of the developed hydrogels. The viability of HUVECs in the Zn@HAs@HN and Zn‐Ga@HAs@HN groups was similar to that in the control group, indicating that the cytotoxicity of the hydrogels was low (**Figure** **4**A). The impact of hydrogels on the angiogenesis of HUVECs was also investigated. Compared to the control group, the wound closure ratio was significantly higher in the Zn@HAs@HN and Zn‐Ga@HAs@HN groups (Figure 4B,D). In addition, the tube formation ability of HUVECs treated with Zn@HAs@HN and Zn‐Ga@HAs@HN hydrogels was also enhanced compared to that of the control group (Figure 4C), as shown by the increased number of junctions, number of meshes, and total segments length (Figure 4E–G). To further evaluate the impact of the Zn‐Ga@HAs@HN hydrogel on angiogenic gene expression, we determined the CD31 and VEGF expression levels in HUVECs in different groups via qRT‒PCR analysis (Figure 4H,J). The results showed that HUVECs treated with Zn@HAs@HN and Zn‐Ga@HAs@HN hydrogels exhibited higher VEGF expression levels.

### 2.5 Effect of Zn‐Ga@HAs@HN Hydrogel on Osteogenesis

**Figure 5:**
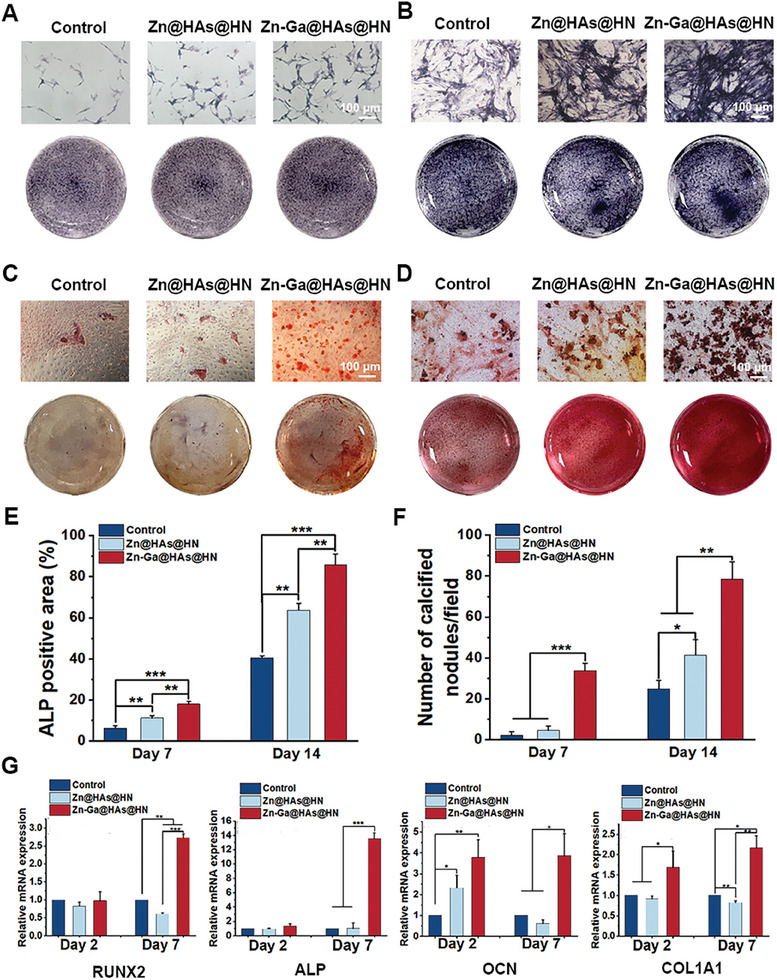
Effects of Zn‐Ga@HAs@HN hydrogel on osteogenesis. A,B) ALP staining results on days 7 (left) and 14 (right). C,D) Alizarin red staining results on days 14 (left) and 21 (right). E) Quantitative analysis of ALP staining results. F) Quantitative analysis of Alizarin red staining results. G) qRT‒PCR analysis of the expression levels of osteogenic‐related markers, including *RUNX2*, *ALP*, *OCN*, and *COL1A1*, in BMSCs on days 2 and 7. (^*^ *p* < 0.05, ^**^ *p *< 0.01, ^***^ *p *< 0.001).

We next investigated the effect of the Zn‐Ga@HAs@HN hydrogel on osteogenic differentiation in vitro. Alkaline phosphatase (ALP) (**Figure** **5**A,B) and Alizarin Red staining (Figure 5C,D) were used to study the effect of the Zn‐Ga@HAs@HN hydrogel on extracellular matrix mineralization. The results indicated that mineral deposition and calcium nodule formation in the Zn‐Ga@HAs@HN hydrogel group were significantly enhanced compared with those in the other groups (Figure 5E,F), suggesting that the Ga^3+^ released from the Zn‐Ga@HAs@HN hydrogel was able to stimulate BMSC osteogenic differentiation. In addition, the expressions of osteogenic‐related genes, including runt‐related transcription factor 2 (*RUNX2*), *ALP*, osteocalcin (*OCN*), and collagen I (*COL1A1*), were determined on days 2 and 7 after osteogenic differentiation culturing (Figure 5G). The results showed that expressions of *OCN* and *COL1A1* increased in the Zn‐Ga@HAs@HN group on day 2 compared with the other groups. On day 7, genes of *RUNX2*, *ALP*, *OCN*, and *COL1A1* were significantly up‐regulated in the Zn‐Ga@HAs@HN group compared with the other groups. These results indicated the excellent osteogenic activity of the Zn‐Ga@HAs@HN hydrogel.

### 2.6 Effects of Zn‐Ga@HAs@HN Hydrogel on Behaviors of RSC96 cells

**Figure 6:**
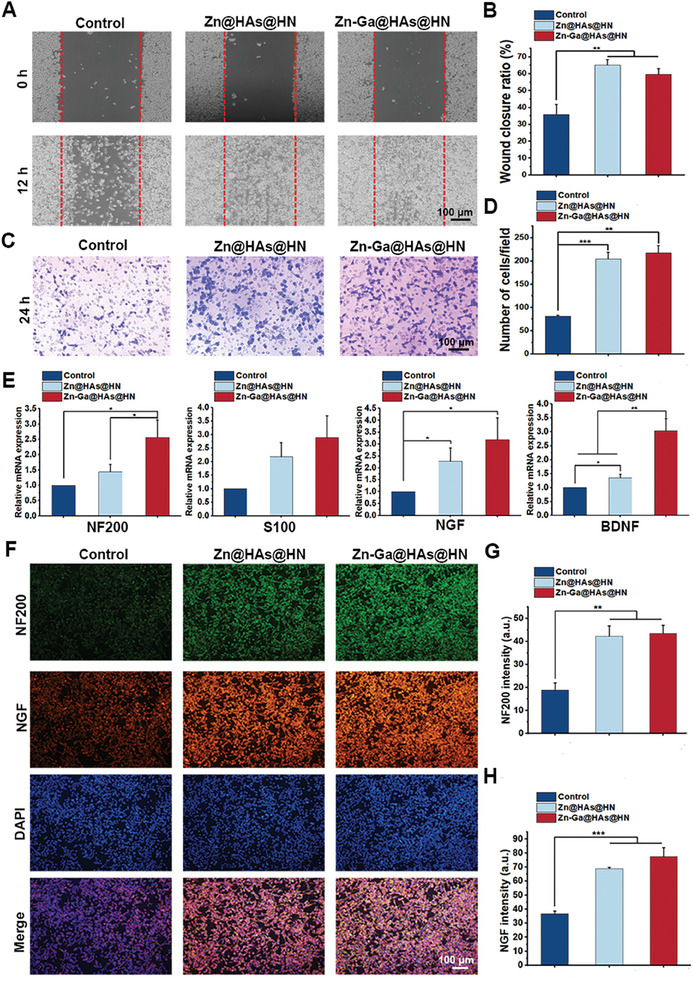
Effects of Zn‐Ga@HAs@HN hydrogel on behaviors of RSC96 cells. A,B) Wound healing assay and corresponding quantitative analysis of RSC96 cells after different treatments. C,D) Transwell assay and corresponding quantitative analysis of RSC96 cells after different treatments. E) qRT‒PCR analysis of the gene expression levels of NGF, BDNF, S100, and NF200 in RSC96 cells after different treatments. F,G) Immunofluorescence staining and corresponding quantitative analysis of NF200 and NGF in RSC96 cells after different treatments. (^*^ *p* < 0.05, ^**^ *p *< 0.01, ^***^ *p *< 0.001).

Based on the effect of HAs on neurogenesis (Figure 1G), we further investigated the impact of Zn‐Ga@HAs@HN hydrogel on the behaviors of RSC96 cells. As shown in **Figure** **6**A, after 12 h of culture, more migrated cells were observed in the Zn@HAs@HN and Zn‐Ga@HAs@HN groups. The migration ratio of RSC96 cells in the Zn@HAs@HN and Zn‐Ga@HAs@HN groups was significantly higher than that in the control group (Figure 6B), suggesting that the released HAs could significantly accelerate RSC96 cell migration. In addition, a Transwell experiment was performed to further confirm the recruitment ability of the hydrogels. More migrated cells were observed in the Zn@HAs@HN and Zn‐Ga@HAs@HN groups than in the control groups, suggesting the superior cell recruitment properties of the Zn@HAs@HN and Zn‐Ga@HAs@HN hydrogels (Figure 6C,D). As shown in Figure 6E, the Zn@HAs@HN and Zn‐Ga@HAs@HN groups exhibited higher expression of NF200, S100, NGF, and BDNF, and there were no significant differences between the Zn@HAs@HN and Zn‐Ga@HAs@HN groups. Furthermore, NF200 and NGF expression levels in RSC96 cells after different treatments were determined via immunofluorescence staining (Figure 6F,G). The results showed that RSC96 cells treated with Zn@HAs@HN and Zn‐Ga@HAs@HN hydrogels exhibited higher fluorescence intensity and expression levels of NF200 and NGF.

### 2.7 Neurogenesis of RSC96 Cells Enhanced Osteogenesis and Angiogenesis

**Figure 7:**
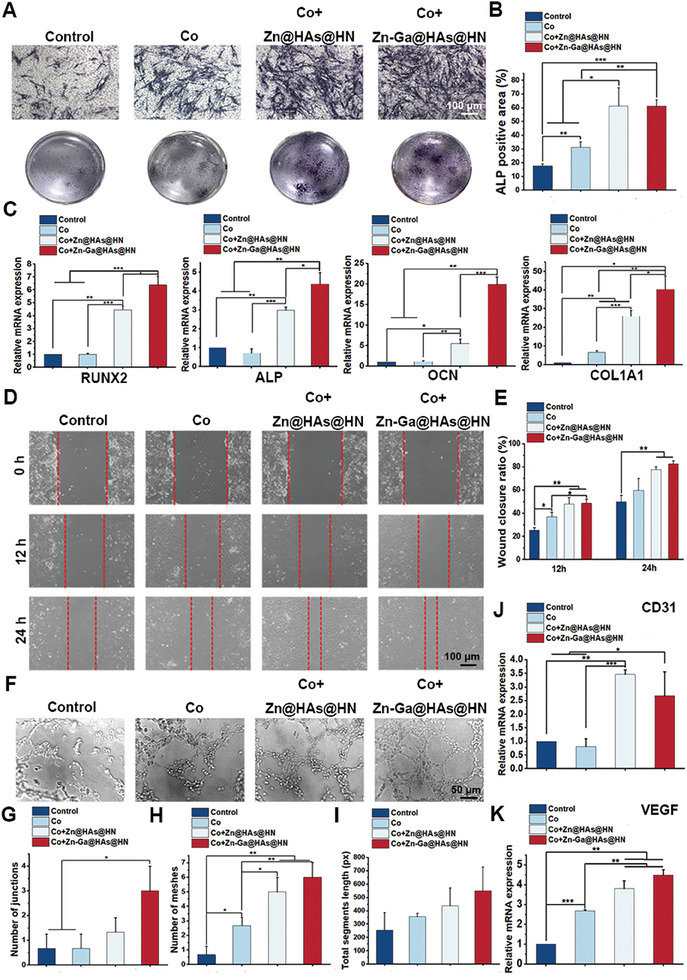
Neurogenesis of RSC96 cells enhanced osteogenesis and angiogenesis. A,B) ALP staining results of BMSCs in different groups. C) qRT‒PCR analysis of the gene expression levels of *RUNX2*, *ALP*, *OCN*, and *COL1A1*. D,E) Wound healing assay of HUVECs in different groups. F–I) Tube formation assay of HUEVCs in different groups. J,K) qRT‒PCR analysis of CD31 and VEGF expression in HUVECs in different groups. Co: coculture. (^*^ *p* < 0.05, ^**^ *p *< 0.01, ^***^ *p *< 0.001).

A cell coculture system was applied to study the effects of neurogenesis of RSC96 cells on the osteogenesis of BMSCs and angiogenesis of HUVECs in vitro. In short, RSC96 cells were placed in the upper chamber and treated with PBS or different hydrogels, while BMSCs or HUVECs were added to the lower chamber. Then, we assessed osteogenic‐ and angiogenic‐related markers after coculture. To study the effect of neurogenesis of RSC96 cells on extracellular matrix mineralization, BMSCs, and RSC96 cells were continuously cocultured for 10 days and then assessed with ALP staining. The results showed that mineral deposition was increased in the Co+Zn@HAs@HN and Co+Zn‐Ga@HAs@HN groups (**Figure** **7**A,B). As shown in Figure 7C, the expression of osteogenesis‐related genes, including *RUNX2*, *ALP*, *OCN*, and *COL1A1*, was significantly increased in the Co+Zn@HAs@HN and Co+Zn‐Ga@HAs@HN groups compared with the other groups. Furthermore, the migration ability of HUVECs was enhanced in the Co+Zn@HAs@HN and Co+Zn‐Ga@HAs@HN groups, as shown by the higher wound closure percentage in the wound healing assay. (Figure 7D,E). The results of the tube formation assay showed that the number of junctions and the number of meshes of HUEVCs were also increased in the Co+Zn@HAs@HN and Co+Zn‐Ga@HAs@HN groups (Figure 7F–I). In addition, the expression of CD31 and VEGF in HUVECs was significantly higher in the Co+Zn@HAs@HN and Co+Zn‐Ga@HAs@HN groups than in the other groups (Figure 7J,K). These results indicated that Zn@HAs@HN and Zn‐Ga@HAs@HN hydrogels were able to promote neurogenesis and then enhance osteogenesis and angiogenesis in turn.

### 2.8 In Vivo Infected Fracture Healing in Mice

**Figure 8:**
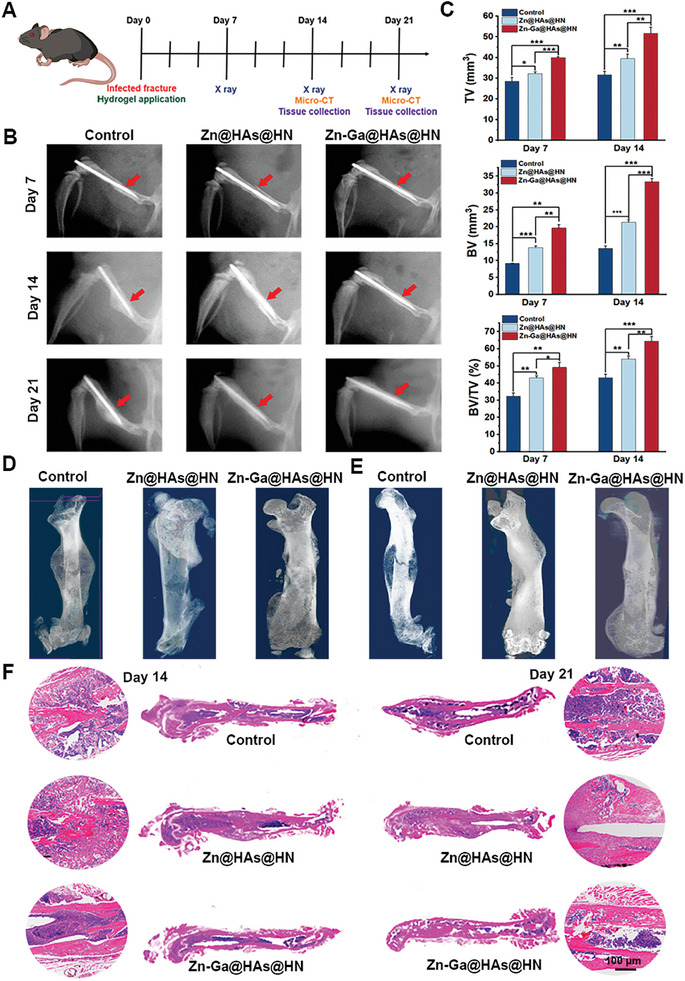
Promotion of infected fracture healing by the Zn‐Ga@HAs@HN hydrogel. A) Schematic illustration of the timeline of the in vivo application of the Zn‐Ga@HAs@HN hydrogel. B) Representative images of X‐ray comparison of the fracture healing process on days 7, 14, and 21. C) Statistical analysis of BV, TV, and BV/TV in each group based on results of micro‐CT. D) Micro‐CT 3D construction images on day 14. E) Micro‐CT 3D construction images on day 21. F) H&E staining of tissues collected on days 14 and 21. (^*^ *p* < 0.05, ^**^ *p *< 0.01, ^***^ *p *< 0.001).

To investigate how the Zn‐Ga@HAs@HN hydrogel affects fracture healing in vivo, we delivered PBS, Zn@HAs@HN hydrogel, and Zn‐Ga@HAs@HN hydrogel to the infected fracture sites in mice. The Zn‐Ga@HAs@HN hydrogel exhibited excellent antibacterial ability in vivo (Figure S5, Supporting Information). The fracture healing process was monitored using X‐rays and microcomputed tomography (micro‐CT) (**Figure** **8**A). As presented in Figure 8B, the fracture gap was significantly reduced in the mice treated with the Zn‐Ga@HAs@HN hydrogel compared with that in the other groups on days 14 and 21. In addition, the Zn‐Ga@HAs@HN hydrogel group exhibited a higher total callus volume (TV), bone callus volume (BV), and value of BV/TV compared to the other groups on days 14 and 21 (Figure 8C; Figure S6, Supporting Information). On day 21, the mice in the control group still exhibited a clear border between hardened callus and cortical bone, which was not observed in the Zn‐Ga@HAs@HN hydrogel group (Figure 8D,E). Hematoxylin and eosin (H&E) staining on days 14 and 21 further indicated increased bone formation area and better bone continuity at the fracture site in the Zn‐Ga@HAs@HN hydrogel group compared to the other groups (Figure 8F). These results showed that the Zn‐Ga@HAs@HN hydrogel effectively accelerated infected fracture healing in vivo.

### 2.9 The Zn‐Ga@HAs@HN Hydrogel Enhanced Angiogenesis, Osteogenesis, and Neurogenesis In Vivo

**Figure 9:**
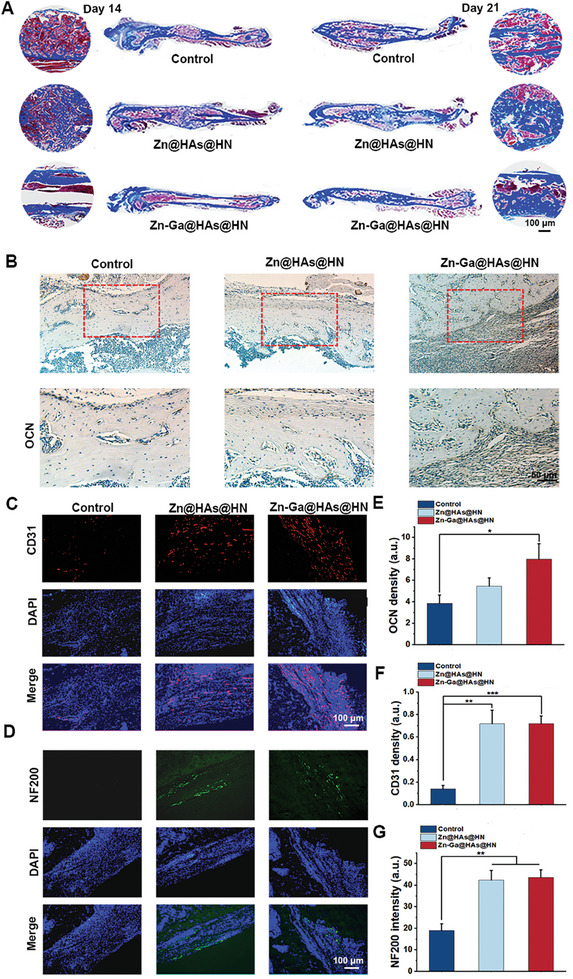
Histological staining for the evaluation of fracture healing. A) Masson's trichrome staining evaluation of bone formation at days 14 and 21. B,E) IHC staining of OCN expression on day 21. C,F) Immunofluorescence staining of CD31 on day 21. D,G) Immunofluorescence staining of NF200 on day 21. (^*^ *p* < 0.05, ^**^ *p* < 0.01, ^***^ *p* < 0.001).

Different degrees of new bone formation were determined using Masson's trichrome staining on days 14 and 21. As shown in **Figure** **9**A, collagen deposition was significantly increased in the fracture sites in the Zn‐Ga@HAs@HN hydrogel group compared to the other groups on days 14 and 21. The results indicated that the Zn‐Ga@HAs@HN hydrogel group exhibited the greatest level of bone formation, while less newly formed bone tissue was observed in the other two groups. In OCN immunohistochemical (IHC) staining, more mature osteoblasts were observed in the Zn‐Ga@HAs@HN hydrogel group than in the other groups (Figure 9B,E), which was in accordance with the results of Masson's trichrome staining. To evaluate the angiogenic ability of the Zn‐Ga@HAs@HN hydrogel in vivo, CD31 immunofluorescence staining was conducted on day 21 (Figure 9C). The results showed that CD31 expression was significantly increased in the Zn@HAs@HN hydrogel and Zn‐Ga@HAs@HN hydrogel groups (Figure 9F), indicating that both Zn@HAs@HN hydrogel and Zn‐Ga@HAs@HN hydrogels displayed a promoting effect on angiogenesis during fracture healing. Furthermore, immunofluorescence staining for NF200 was carried out to assess nerve formation in the fracture sites. The results showed that the Zn@HAs@HN hydrogel and Zn‐Ga@HAs@HN hydrogel groups had a higher intensity of green fluorescence, indicative of enhanced neurogenesis (Figure 9D,G).

## 3 Discussion

Infection could affect the physiological process and hamper fracture healing.^[^ ^26^ ^]^ Infection‐induced bone nonunion seriously affects the quality of life of patients and poses serious challenges for society and public health systems.^[^ ^27^ ^]^ Currently, hydrogel dressings are widely used for promoting fracture healing and bone regeneration owing to their excellent biocompatibility, biodegradability, and drug‐delivering capacity.^[^ ^28^, ^29^ ^]^ However, the application of hydrogels in bone fracture healing is still limited by limited bioactivity and a lack of antibacterial capacity.^[^ ^30^ ^]^ In our previous study, we fabricated an in situ‐forming hyaluronic acid‐catechol‐NCSN hydrogel, which exhibited promising bioadhesiveness and offered a feasible and effective therapeutic approach for tissue regeneration.^[^ ^31^ ^]^ In the present study, we developed the microenvironment‐sensitive Zn‐Ga@HAs NPs to release the Zn^2+^, Ga^3+^, and HAs according to the pH value in the fracture sites to improve the utilization efficiency and reduce side effects. The Zn‐Ga@HAs NPs directly exert an inhibition effect on bacteria growth. As shown by in vitro results, the Zn‐Ga@HAs NPs had good antibacterial, anti‐inflammatory, and antioxidant abilities, indicating that the hydrogel could alleviate infection, inflammatory response, and oxidative damage during the early stage of fracture healing. During the late stage, through controlled and sustained release of Zn^2+^, Ga^3+^, and HAs, the hydrogel fully meets the requirements of bone regeneration by enhancing angiogenesis, osteogenesis, and neurogenesis. Furthermore, HAs were able to scavenge ROS and alleviate the damage caused by the Zn^2+^ and Ga^3+^. Therefore, our study demonstrated that the developed Zn‐Ga@HAs@HN hydrogel showed great application promise for treating infected bone fractures.

Although great progress has been made in treating bone fractures in the past decades, few researchers have fully considered nerve regeneration during fracture healing. Recently, emerging evidence has indicated that crosstalk between bone and nerves plays an important role in bone repair. Generally, sensory nerves and the sympathetic nervous system (SNS) are proposed to penetrate the cortical bone parallel to the blood vessels.^[^ ^32^ ^]^ Following fracture, the peripheral nervous system interrupts synapses and switches to a regenerative state, promoting bone regeneration through neuropeptides or neurotransmitters, including calcitonin gene‐related peptide (CGRP), substance P, norepinephrine, acetylcholine, neuropeptide Y, and neurotrophins.^[^ ^10^, ^12^ ^]^ Recent findings also suggest that NGF is important to BMSC migration and osteogenesis, as well as revascularization during early bone repair.^[^ ^33^, ^34^, ^35^ ^]^ Thus, improving nerve regeneration and function during fracture healing will facilitate bone regeneration and accelerate fracture healing. In this study, we first revealed that HAs significantly increased neurogenic gene expression in RSC96 cells. The enhanced neurogenesis further promoted BMSC osteogenesis and HUEVC angiogenesis. The in vivo experiments confirmed that bone formation, vessel growth, and nerve regeneration were enhanced in the Zn‐Ga@HAs@HN groups. Furthermore, we identified four signaling pathways (Figure 1F), including Notch, ERBB, JAK‐STAT, and sphingolipid signaling pathways, that were altered after HAs treatments in RSC96 cells. It has been demonstrated that the Notch,^[^ ^36^ ^]^ ERBB,^[^ ^37^ ^]^ JAK‐STAT,^[^ ^38^ ^]^ and sphingolipid,^[^ ^39^ ^]^ signaling pathways are involved in the regulation of neurogenesis. Thus, we hypothesized that HAs might induce neurogenesis of Schwann cells via these signaling pathways. Further investigation is needed to fully uncover the underlying mechanisms.

Bone fracture healing is a dynamic process that includes four continuous phases: the inflammatory phase, soft callus formation phase, hard callus formation phase, and remodeling phase.^[^ ^40^ ^]^ It should also be considered that different fracture healing phases require different molecular stimuli. It has been demonstrated that the pH value in different phases is different. During the early healing phase, insufficient blood supply and accumulation of acidic metabolites result in reduced pH at the fracture site. Subsequently, the pH increases to more alkaline values with the process of fracture healing.^[^ ^41^ ^]^ In this study, the pH‐responsive Zn‐Ga@HAs@HN hydrogel successfully eradicated bacterial infection and alleviated oxidative damage in the early healing phase, and released Zn^2+^, Ga^3+^, and HAs to promote bone regeneration in the later stage. The controlled release of Zn^2+^, Ga^3+^, and HAs according to pH at the fracture site reduced toxicity and provided optimized therapeutic effects. Herein, we mainly utilized the hydrogel as a drug carrier to delivery Zn^2+^, Ga^3+^, and HAs into the fracture site. The mechanical properties of hydrogel, such as the strength and modulus, also impact fracture healing. However, hard scaffolds often exhibit relatively slow degradation rates. It is necessary to develop hydrogels with satisfactory mechanical strength and drug delivery behavior for fracture healing.

These results showed that the Zn‐Ga@HAs@HN hydrogel is a promising drug delivery system to promote infected fracture healing with synergistic regulation of angiogenesis, osteogenesis, and neurogenesis. However, the present study still has some limitations. First, Using antibiotics as a positive control would support our conclusion. Second, it has been demonstrated that Type H vessel, a specific subtype of bone vessels, promote fracture healing.^[^ ^42^ ^]^ The effect of Zn‐Ga@HAs@HN hydrogel on Type H vessel formation needs to be further investigated. Third, the in vivo degradation behavior of the hydrogel is also important. The residence time of hydrogels in situ needs to be determined.

## 4 Conclusion

To improve drug‐resistant bacterial‐infected fracture healing, we fabricated a nanoarchitecture‐integrated Zn‐Ga@HAs@HN hydrogel by mixing the Zn‐Ga@HAs NPs and the polymer HN. The pH sensitivity of Zn‐Ga@HAs NPs enables the controlled release of Zn^2+^, Ga^3+^, and HAs in the fracture microenvironment according to pH value. In the early stage, the Zn‐Ga@HAs NPs eliminated bacterial infection, reduced oxidative stress, and created a pro‐healing microenvironment for tissue regeneration. In the later stage, the released Zn^2+^, Ga^3+^, and HAs promoted angiogenesis–osteogenesis–neurogenesis tripling. The cytokines NGF and BDNF from new nerve cells further enhanced angiogenesis and osteogenesis. Ultimately, a mutually supporting angiogenesis–osteogenesis–neurogenesis cycle formed at the fracture site. These findings highlight the promising application prospects of the Zn‐Ga@HAs@HN hydrogel for the treatment of drug‐resistant bacteria‐infected fracture healing.

## Conflict of Interest

The authors declare no conflict of interest.

## Author Contributions

K.Z., W.H., Y.X., and S.Z. contributed equally to this work. K.Z., W.H., Y.X., and S.Z. conducted experiments and wrote the manuscript; M.T., P.B., and Y.Z. developed the figures; W.Z., Z.L., and Y.H. edited the manuscript; MA.S., Q.F., G.L., and B.M. conceived and supervised the study and revised the manuscript. All the authors have read and approved the final manuscript.

## Supporting information

Supporting Information

## Data Availability

The data that support the findings of this study are available on request from the corresponding author. The data are not publicly available due to privacy or ethical restrictions.
