# Supplementary material for: Nanoarchitecture‐Integrated Hydrogel Boosts Angiogenesis–Osteogenesis–Neurogenesis Tripling for Infected Bone Fracture Healing
Source: Adv Sci (Weinh). 2024 Sep 5;11(43):2406439. doi: 10.1002/advs.202406439 (PMC11578348; doi:10.1002/advs.202406439)
Supplement: Supplementary file 1 — Supporting Information [file ADVS-11-2406439-s001.docx]

**Supporting Information**

**Nanoarchitecture-Integrated Hydrogel Boosts Angiogenesis-Osteogenesis-Neurogenesis Tripling for Infected Bone Fracture Healing**

*Kangkang Zha*, *Weixian Hu*, *Yuan Xiong*, *Shengming Zhang*, *Meijun Tan*, *Pengzhen Bu*, *Yanzhi Zhao*, *Wenqian Zhang*, *Ze Lin*, *Yiqiang Hu*, *Mohammad-Ali Shahbazi**, *Qian Feng**, *Guohui Liu**, *Bobin Mi**

**1. Supplementary figures**


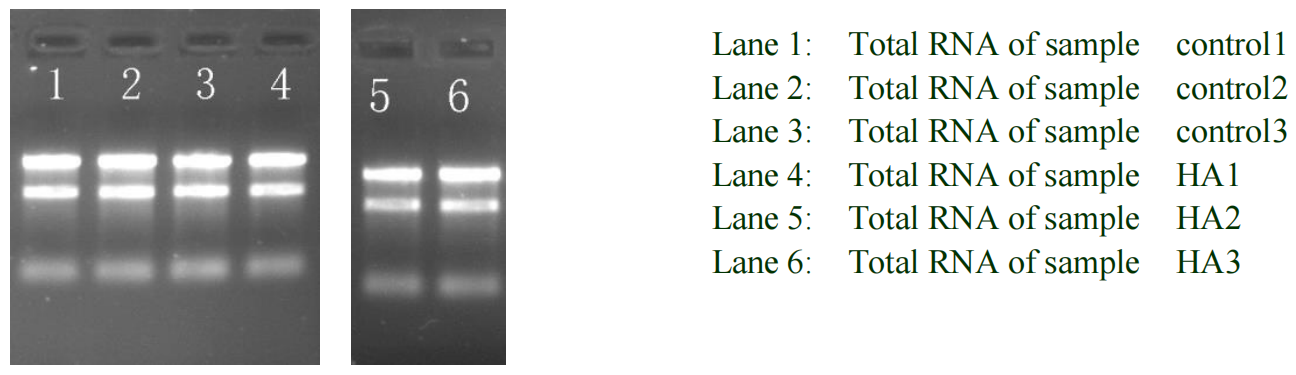


**Figure S1.** RNA integrity and gDNA contamination test by denaturing agarose gel electrophoresis.


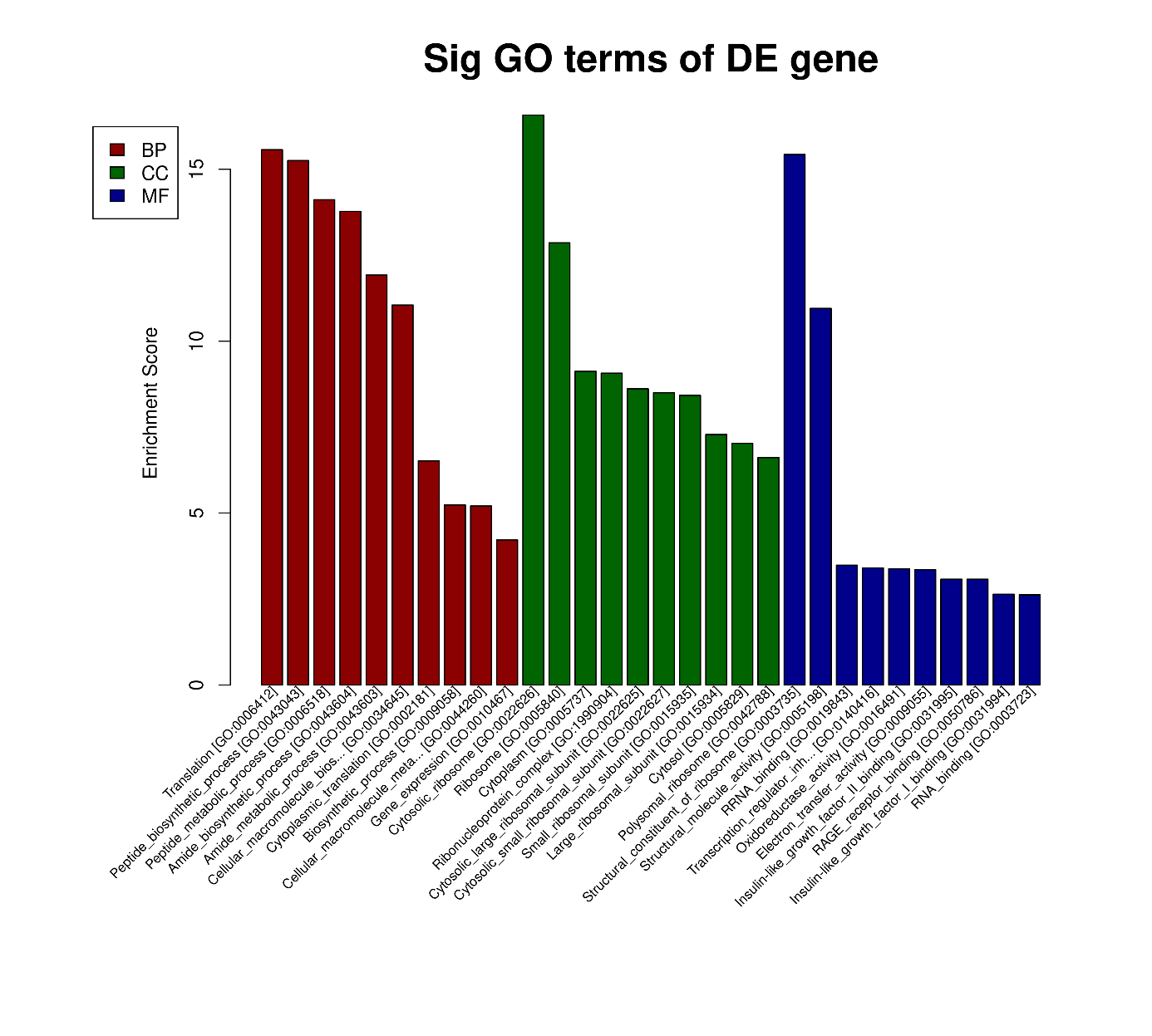


**Figure S2.** GO classification of genes in RSC96 cells before and after treatment with HAs


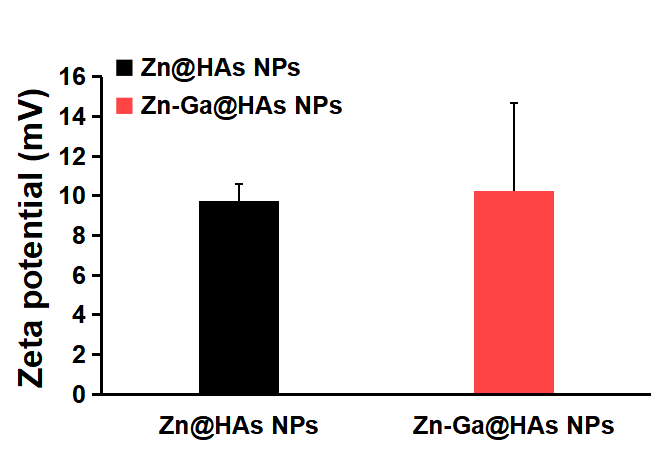


**Figure S3.** Zeta potential of Zn@HAs NPs and Zn-Ga@HAs NPs.


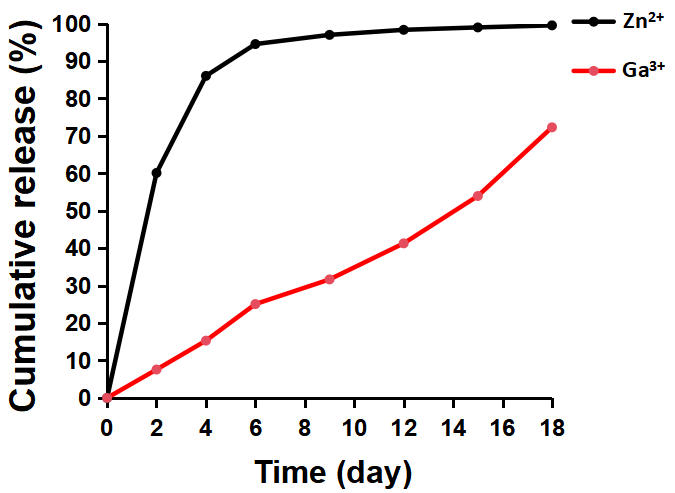


**Figure S4.** Release of Zn^2+^ and Ga^3+^ from the Zn-Ga@HAs@HN hydrogel in PBS at 37 ℃.


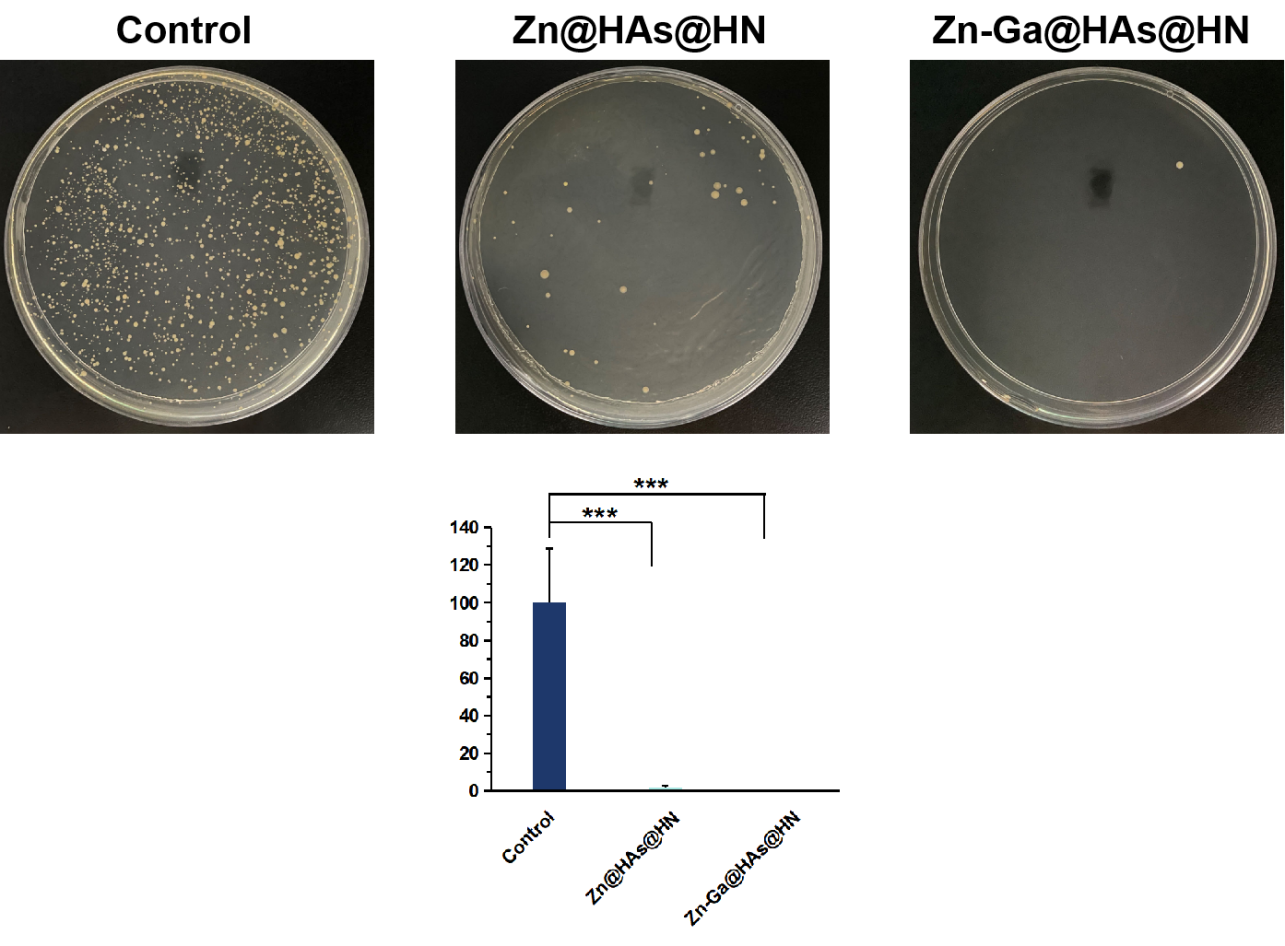


**Figure S5.** In vivo antibacterial activity of the hydrogels.


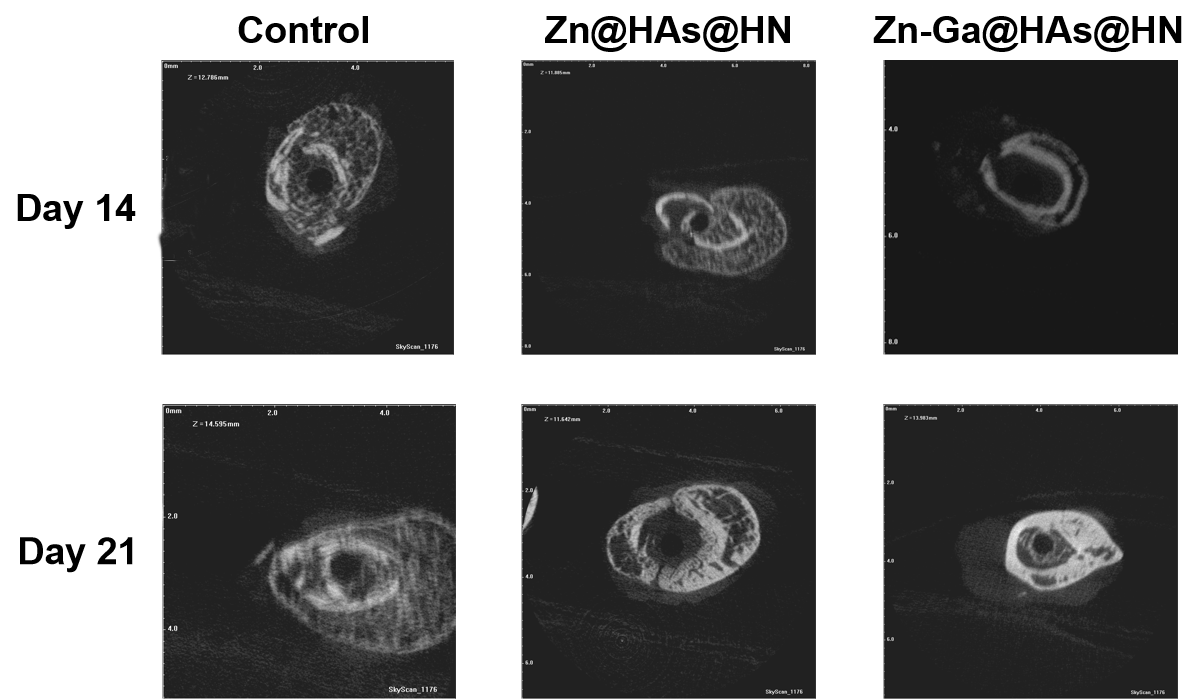


**Figure S6.** Cross section images of micro-CT on post-fracture days 14 and 21.

**2. Experimental Methods**

**2.1. Animals**

C57BL/6J mice were purchased from Hubei Biont Biotechnology Co., Ltd., and this study was performed according to the guidelines of the Animal Care and Use Committee of Tongji Medical College, Huazhong University of Science and Technology (approval number for animal experiments: 3921).

**2.2. RNA-seq**

Total RNA was isolated from RSC96 cells treated with 0.2 mg/mL HAs or PBS (control) using TRIzol reagent. The RNA concentration was measured using the NanoDrop ND-1000 Assay Kit, followed by RNA enrichment by oligo (dt). The RNA library was established using the KAPA Stranded RNA-Seq Library Prep Kit and sequenced using an Illumina NovaSeq 6000 sequencer. The raw sequencing data was quality controlled before quantitative analysis. GO gene set analysis, KEGG pathway enrichment analysis, and GSEA were performed to determine the differentially expressed genes and altered signaling pathways in the two groups.

**2.3. Western blot analysis**

Total cellular proteins were extracted using RIPA solution and determined by BCA assay. Then, 30 μg of total protein were loaded onto SDS gels, separated by electrophoresis for 80 min, and then transferred onto PVDF membranes for 50min. Finally, they were blocked with 5% (w/v) skimmed milk at room temperature for 1 h and incubated with primary antibodies overnight at 4 °C. After washing with TBST thrice, the membranes were incubated with the secondary antibodies for 60 min at room temperature. Then the membranes were treated with ECL chemiluminescent substrate and visualized using a ChemiDoc MP chemiluminescence gel imaging system.

**2.4. Synthesis of Zn-Ga@HAs NPs**

First, ZnCl_2_ (0.5 g) and Ga(NO_3_)_3_ (0.5 g) were dissolved in 30 mL distilled water. Then, HAs (1.0 g) was added to the above solution. After 4 h of reaction, 0.1 M HCl was added to the solution to adjust the pH to 5. Next, the solution was centrifuged, and the precipitate was collected. The products were dried in a vacuum chamber for 48 h.

**2.5. Preparation of** **Zn-Ga@HAs@HN hydrogel**

The HA-NCSN was synthesized as described in previous study ^[28]^. Briefly, HA (1 g) was dissolved in 200 mL distilled water and added with N-(3-dimethylaminopropyl)-N’-ethylcarbodiimide hydrochloride (2.396 g), HOBt (1.914 g), and adipic dihydrazide (ADH) (34.84 g) to obtain HA-ADH. Next, methyl isothiocyanate (5 g) was added to the 20 mL HA-ADH solution (5%, w/v), and stirred for 3 days to obtain HA-NCSN. Then, 10% HA-NCSN (w/v) and Zn-Ga@HAs (40 mg/mL) were thoroughly mixed and stirred vigorously. Then, the pH of the solution was adjusted to 9 by the addition of 0.1 M NaOH to form the Zn-Ga@HAs@HN hydrogel.

**2.6. Characterization of Zn-Ga@HA NPs and Zn-Ga@HA@HN hydrogels**

The morphologies of the Zn@HAs NPs, Zn-Ga@HAs NPs, Zn@HAs@HN hydrogel and Zn-Ga@HAs@HN hydrogel were observed by SEM (LIBRA 200 CS, Carl Zeiss Co., Germany). To assess the swelling ratio of the hydrogel, the freeze-dried Zn@HAs@HN and Zn-Ga@HAs@HN hydrogels were weighed (W_0_) and then incubated in PBS at 37 °C. At different time points, the weights of the hydrogels were measured (W_x_). The swelling ratio was calculated as follows: Swell ratio (%) = (W_x_ − W_0_)/W_0_ × 100. To evaluate the degradation rates of the hydrogels, Zn@HAs@HN and Zn-Ga@HAs@HN hydrogels were immersed in PBS and incubated at 37 ℃. The hydrogel weights were measured at different time points. The degradation rate was calculated using the following equation: Degradation rate (%) = (W_0_-W_t_)/W_0_ × 100, where W_0_ represents the weight on day 0, and W_t_ represents the weight at different time points. The concentrations of Zn^2+^ and Ga^3+^ released from the Zn-Ga@HAs@HN hydrogel were detected by ICP-OES (Agilent, USA). Compression tests were performed using a MACH-1 Micromechanical System (Biomomentum Inc., Canada).

**2.7. *In vitro* antibacterial activity**

To investigate the antibacterial properties of the composite hydrogels, 1 mL of MRSA (1 × 10^7^ CFU/mL) bacterial solution was incubated with 100 μL of Zn@HAs@HN and Zn-Ga@HAs@HN hydrogels or PBS for 12 h. Then, the bacterial supernatant was collected, and 100 μL of the supernatant was spread on agar plates. The bacteria were incubated at 37 °C for 18 h to observe the colonies. Next, we performed an inhibition zone experiment to further evaluate the antibacterial properties of the developed hydrogels. Briefly, 100 μL of MRSA suspension (1 × 10^8^ CFU/mL) was spread on agar plates. Then, the filter paper soaked with hydrogels or PBS was placed on the center of the plate and incubated at 37 °C for 24 h. The area with no bacterial colonies was measured.

**2.8. Antioxidant assay**

HUEVCs were seeded in 96-well plates at a density of 2 × 10^4^ cells/well and treated with 500 μM H_2_O_2_, followed by treatments with the extracts of different hydrogels or PBS for 6 h. Then, the intracellular ROS levels were measured by staining with a DCFH-DA probe for 20 min at room temperature. For cytoskeleton staining, the cells were fixed with 4% paraformaldehyde, washed with PBS, and incubated with phalloidin (Beyotime) for 30 minutes at room temperature, followed by DAPI staining. All samples were observed and captured with a microscope (IX53, Olympus).

**2.9. *In vitro* cytocompatibility**

HUEVCs were seeded in 96-well plates at a density of 2 × 10^3^ cells/well and treated with 10 μL hydrogel extracts. The cells were incubated in DMEM/F12 containing 10% FBS at 37 °C. The viability of HUEVCs was assessed by a Calcein AM/propidium iodide (PI) live-dead staining kit (Solarbio, Beijing, China). Images were obtained with a fluorescence microscope (Olympus).

**2.10. Wound healing assay**

HUVECs or RSC96 cells were seeded in 24-well plates at a density of 2 × 10^5^ cells/well. After reaching 90% confluence, wounds were made in each well with a sterile 200 μL pipette tip, and the cells were washed twice with PBS. Then, the cells were treated with the extract of different hydrogels or PBS and cultured with DMEM/F12 containing 1% FBS. The scratches were observed at 12 h and 24 h with an optical microscope (Olympus). The wound closure ratio was assessed using ImageJ software.

**2.11. Tube formation assay**

To investigate the angiogenic effect of the composite hydrogels, HUVECs were seeded in 6-well plates and treated with the extracts of different hydrogels for 24 h. Then, the cells (2 × 10^4^/well) were seeded in 96-well plates precoated with Matrigel (Corning, NY, USA) and cultured for 6 hours. The tubular structure was observed and captured with an inverted microscope (Olympus). The images were analyzed by ImageJ software.

**2.12. BMSC osteogenic differentiation**

BMSCs were seeded in 24-well plates at a density of 8 × 10^4^/well and continuously cultured in osteogenic medium to induce osteogenesis. On days 7 and 14, an ALP staining kit was used to evaluate the ALP activity according to the instructions. The cells were stained with BCIP/NBT substrate for 24 h. On days 14 and 21, 0.5% alizarin red staining dye was used to observe the formation of mineralized nodules. All images were obtained with an optical microscope (Olympus) and analyzed by ImageJ software.

**2.13. Migration assay**

Migration was evaluated using a 24-well Transwell system including 8 μm pores. RSC96 cells (2 × 10^4^/well) in 100 μL 1640 medium without FBS were seeded in the upper chamber, and 1640 medium with 100 µL hydrogels was added to the lower chambers. After incubation for 24 h, the upper chambers were cleaned with cotton applicators to remove the cells, and the cells in the lower chambers were fixed with 4% paraformaldehyde. The samples were stained with 0.1% crystal violet for 30 min and photographed under an optical microscope (Olympus, Japan).

**2.14. Immunofluorescence staining**

After being treated with different hydrogels for 12 h, the RSC96 cells were washed twice in PBS and then fixed in 4% paraformaldehyde (PFA) for 15 min, followed by blocking for 30 min in 1% bovine serum albumin (BSA) at room temperature. Then, the cells were incubated with anti-NF200 antibody and anti-NGF antibody at 4 ℃ overnight. After being rinsed with PBS, the samples were incubated with secondary antibody for 1 h at room temperature. Finally, the cells were stained with DAPI for 5 min. The images were photographed with a fluorescence microscope (Olympus, Japan) and analyzed by ImageJ software.

**2.15. qRT‒PCR analysis**

RNA was extracted using RNA-easy Isolation reagent (Vazyme Biotech, Nanjing, China) according to the manufacturer’s instructions. RNA was converted to cDNA using a HiScript® III RT SuperMix kit (Vazyme Biotech, China), and gene expression was measured by qRT‒PCR using ChamQ SYBR qPCR Master Mix (Vazyme Biotech, China) and a CFX96 Real-Time PCR Detection System. All primers were listed as follows: NF200-forward: GTTCCGAGTGAGGTTGGACC, NF200-reverse: CCGCCGGTACTCAGTTATCTC; S100-forward: AGAGGGTGACAAGCACAAGC, S100-reverse: TCCTGCTCTTTGATTTCCTCCAG; NGF-forward: GGCCACTCTGAGGTGCATAG, NGF-reverse: CTATCTGTGTACGGTTCTGCC; BDNF-forward: AAGTCTGCATTACATTCCTCGA, BDNF-reverse: GTTTTCTGAAAGAGGGACAGTTTAT; RUNX2-forward: CGCCACCACTCACTACCACAC, RUNX2-reverse: TGGATTTAATAGCGTGCTGCC; ALP-forward: CCAACTCTTTTGTGCCAGAGA, ALP-reverse: GCTACATTGGTGTTGAGCTTTT; OCN-forward: TTCTGCTCACTCTGCTGACCC, OCN-reverse: CTGATAGCTCGTCACAAGCAGG; COL1A1-forward: CTGACTGGAAGAGCGGAGAG, COL1A1-reverse: CGGCTGAGTAGGGAACACAC; Rat β-actin-forward: ATCATTGCTCCTCCTGAGCG, Rat β-action-reverse: GAAAGGGTGTAAAACGCAGCTC; Human β-actin-forward: CATGTACGTTGCTATCCAGGC, Human β-action-reverse: CTCCTTAATGTCACGCACGAT; Mouse β-actin-forward: GGCTGTATTCCCCTCCATCG, Mouse β-action-reverse: CCAGTTGGTAACAATGCCATGT.

**2.16. *In vivo* infected bone fracture healing**

The mice (male, 8 weeks old) were anesthetized with an intraperitoneal injection of pentobarbital sodium (50 mg/kg), and a model of a femoral fracture was made via longitudinal incision. The femur was cut using a diamond disk to yield a mid-diaphysis fracture line. Then, the fractured bone was stabilized by a 25-gauge intramedullary needle. Each fracture site was infected with 10 μL of MARSA suspension (1 × 10^8^ CFU/mL) for 10 min. Then, PBS, HAs, Zn@HAs@HN hydrogel, and Zn-Ga@HAs@HN hydrogel were applied to the fracture sites (n=10). In each fracture site, 50 uL of PBS or hydrogel was directly implanted. To secure the hydrogel, we strive to minimize tissue damage during tissue dissection and sutured the surrounding fascia and muscle carefully. On day 3, the tissue in the fracture callus was collected for in vivo antibacterial assay. On days 14 and 21, half of the mice were sacrificed, and the bone tissues were collected for further analysis.

**2.17. X-ray examination**

On days 3, 7, 14, and 21 post fracture, the mice were observed with X-ray by an In-Vivo FX PRO imaging system (BRUKER, Karlsruhe, Germany). The exposure time was set to 30 s.

**2.18. Micro-CT examination**

On days 14 and 21, the harvested bone tissues were scanned using the micro-CT system (BRUKER 1276, Karlsruhe). The reconstructed (3D) images were generated using CT-Vox version 2.1 software. Parameters including total volume (TV), bone volume (BV), and BV/TV were analyzed with CTAN version 1.12 software.

**2.19. Histologic analysis**

The harvested bone tissues were fixed with 4% paraformaldehyde and then demineralized with decalcification fluid for three weeks. Hematoxylin−eosin (H&E) and Masson’s trichrome staining were performed to assess new bone formation in the fracture sites. Furthermore, immunohistochemistry (IHC) was carried out to determine the OCN expression level. Angiogenesis and neurogenesis were investigated by immunofluorescence staining. CD31 was selected as a marker for vessel formation, and NF200 was selected as a marker for nerve formation. Sections were photographed by an IX53 microscope (Olympus), and the statistical analysis was performed using ImageJ software.

**2.20. Statistical analysis**

The data are shown as the mean ± standard deviation (SD). The results were analyzed with Student’s t test and one-way ANOVA by SPSS 26.0 software. A value of p < 0.05 was considered statistically significant.
